# Perception of general dental practitioners toward periodontal treatment: A survey

**DOI:** 10.4103/0972-124X.44086

**Published:** 2008

**Authors:** Amita Mali, Rohini Mali, Hardik Mehta

**Affiliations:** Department of Periodontology, Bharati Vidyapeeth Dental College and Hospital, Pune – 411 043, Maharastra, India

**Keywords:** General dental practitioner, periodontal, survey

## Abstract

Periodontology is a fast evolving field where newer insights into existing concepts are changing the face of the traditional periodontal treatment. Constant research is taking place so as to develop a number of newer avenues in the treatment of the periodontal diseases. However, the protocol of management of periodontal diseases in the setup of general dental practice has undergone little change over the last decade. So, a survey was carried out among 100 general dental practitioners by means of a questionnaire to identify the current status of periodontal treatment in dental clinics, the protocol of maintenance therapy, and the general awareness of the dental profession toward periodontal care.

## INTRODUCTION

The speciality of periodontology is evolving in all aspects ranging from newer advances in diagnosis, to the use of growth factors and regenerative techniques in treatment. These evidence-based advances have given periodontal diagnosis and treatment a level of predictability of success, which was lacking just a decade ago. Quality dental institutes, which incorporate all these advances in their setup and curriculum have evolved all over the country. Thus, today we see periodontology as a speciality reaching newer heights, and with a very bright future in front of us.

Inspite of the developing of quality dental institutes, a majority of the population visits private dental clinics for their dental needs, especially in the urban areas. The development of internet as a reliable source of knowledge, and the publication of quality journals such as JISP and JIDA has made knowledge of periodontal treatment freely accessible to all practicing dentists. However, it is a common complaint of periodontists that the knowledge of periodontal diagnosis and treatment in the minds of a general dentist is sometimes limited to the level taught in the BDS curriculum at the time of their graduation.

There is a need to evaluate the attitude and perception of the general dental practitioners toward periodontal treatment, as they form the cornerstone of dental practice. Hence, this study aims to identify the various aspects of periodontal treatment provided at a general dental clinic, along with referrals to periodontists.

This study, by the means of a questionnaire, aims to identify the current status of periodontal treatment in clinics, the protocol of maintenance therapy, and the general awareness of the dental profession toward periodontal care.

The evaluation of the status of periodontal care in private dental setups in urban regions of India has not been done before. Thus, this study is the first of its kind in India. Within dentistry, a limited body of literature exists with regard to status of periodontal care, and referral relationships. Many of the studies conducted in this area have attempted to analyze the various aspects of the relationship between the referring doctor and specialist. They have compiled the opinions and observations of both referring general practitioners (GPs) and specialists regarding periodontal care.[1–4]

In a study of a similar pattern, Zemanovich *et al*, have evaluated the demographic variables affecting patient referrals from general dental clinic to a periodontist. They concluded that various factors such as gender of the dentist and the proximity to a periodontist affected the number of referrals by a general dentist.[5] Also, treatment of chronic periodontal disease was the most common periodontal procedure that was referred, followed by soft tissue grafting procedures, and implants. A comprehensive demographic study in the United States was authorized by the American Academy of Periodontology (AAP) in 1981. The study concluded that GPs, in the prime of their careers tend to be the best source of referrals for periodontists.[6] Our study intends to identify the patterns of referrals from general dental practitioners to periodontists.

## MATERIALS AND METHODS

The study was carried out in the form of a survey among 100 general dental practitioners having their dental clinics in the urban areas of Pune city. A questionnaire (Appendix I) comprising of eight questions was distributed to each of them by visiting their clinic and collected on the next day. The questions ranged from the chief periodontal complaints of the patients, to the level of satisfaction of private dentists after periodontal therapy. The questionnaire was prepared by mutual discussion among the examiners. The questionnaires used by previous studies[7–10] were used as a starting point for discussion. They were then adapted according to the prevailing Indian conditions. Finally, a questionnaire consisting of eight questions and subquestions was prepared. This has been attached as an appendix.

Dental practitioners with a dental clinic in an urban area, minimum qualification of BDS, and experience of at least one year in private clinical setup were included for the study.

Interns, dental students, dentists exclusively working in a dental institute and periodontists were excluded from the study.

### Statistical analysis

For each question, independent percentage was calculated to determine the frequency of the responses. To identify the variable factor affecting the responses, multivariate logistic regression analysis test was used.

## RESULTS AND DISCUSSION

Of 100 dentists included, 64 were males while 36 were females. Average years of experience were 5.2 years ranging from 1.6–34 years. The results revealed that a whopping 98% of the GPs performed phase-I therapy on their own and all of them carried out scaling, advised use of mouth washes, and proper brushing techniques. More than two-thirds of the dentists did not carry out splinting at all. Thus, temporary or permanent splinting does not have a high degree of acceptability in private dental clinics.

Nonsurgical periodontal therapy is gaining equal amount of importance across the world as the surgical therapy. However, our survey indicates that the role of a periodontist in private dental clinics in Pune is chiefly limited to surgical therapy. Root planing as a procedure was not carried out by majority of dentists. However, a positive finding from this question was that there is high awareness among dentists regarding demonstration of proper oral hygiene maintenance methods.

Thirty eight percent of the dentists carried out some or the other periodontal surgical procedures on their own. Gingivectomy was the most common procedure carried out by general dentists followed by flap surgery, crown lengthening, and ridge augmentation. Hardly any dentists performed mucogingival surgeries or bone grafting/GTR procedures.

Almost half of the dentists referred their patients to a periodontist for surgical treatment and half of them referred them once a month while a very few of them referred patients twice a week. Though the cardinal sign of periodontal disease is considered to be the periodontal pocket/loss of attachment, in our study we found that only one in every three dentists surveyed referred patients to a periodontist based upon the presence of pockets.

Another interesting response to be noted is that almost half the number of dentists referred patients to a periodontist for chief complaint of mobile teeth. Also, answers to later questions revealed that a similar percentage, that is, 54% of the dentists believe that there is only an occasional reduction in mobility following periodontal treatment. This finding gives us an indication that increasing research should be focused on the satisfactory management of mobile teeth.

Flap surgery, along with bone grafting/GTR is the cornerstone of periodontal practice in general dental clinics. Surprisingly, less number of dentists referred their patients to a periodontist for root coverage procedures. Still a lot of GP's don't believe that implants are an integral aspect of periodontist's armour.

Asked about the success of the periodontal treatment, almost all of them believed that there was stoppage of bleeding after the treatment, while two-thirds of dentists believed that there is occasional recurrence after flap surgery and there is no success with root coverage procedure. Thus, there is high level of satisfaction with the treatment of the most common periodontal complaints. There is no consensus among general dentists that periodontal treatment always increases the lifespan of teeth. Lack of maintenance and awareness toward oral hygiene measures was identified by the survey as the chief factor for recurrence of periodontal disease.

Half of the dentists recalled patients after three months of surgical treatment. Though recall was made after three months many dentists commented that patient's compliance was a problem.

General dentists believed that surgical periodontal treatment remains unaffordable to a large section of our population. Cost structure and methods of payment may differ in different cities and even in different areas of the same city.

Based upon the results of this survey, we have identified certain factors which could be changed for the betterment of the society. These factors are grouped as periodontist factor, general dentist factor, and patient's factor.

### Scope for periodontist

There is a need to increase predictability of various root coverage procedures. There is also a need to develop indigenous graft materials and membranes which would be economical to both the patient and the dentist, and maybe, more acceptable. If affordability of periodontal treatment increases in private setups, larger cross-section of patients would be willing to undergo periodontal treatment.

### Scope for general dentist

The general dentist should be emphasized the importance of regular gingival examination along with probing. He should also be convinced about the important role he plays in motivating the patients and devising a proper protocol for recall. He should be explained the benefits of involvement of periodontist in phase-I therapy and about the important role that a periodontist plays in multidisciplinary dentistry. Seminars and courses should be conducted so as to impart knowledge regarding root coverage procedures.

### Scope for patient

Patients should be made aware regarding the maintenance of oral hygiene measures. They should be convinced and motivated regarding the value of the periodontal treatment compared to its cost. They should be explained that periodontal treatment enhances the longevity of his dentition and provides him with increased treatment options.

### Future directions

Such studies should be performed in future to get an overall perception of the general dentists toward periodontal treatment in India. Such studies performed at different intervals of time in the same cities can also provide an idea about the changing trends and pattern of dental treatment performed by the general dental practitioners. Such studies can also flash a warning light, and force the general dentist and the periodontist to introspect for the betterment of the speciality.

## Appendix I

### Questionnaire

          Date:

1. Name:

2. Total no. of Years of practice:

3. Do you refer your patients to a periodontist for phase-I^*^ therapy?

☐ YES     ☐ NO

A. If NO

Which periodontal therapy do you carry out in your clinic?

- Scaling
- Scaling and root planing
- Advice proper brushing technique
- Advise mouthwashes
- Advise stoppage of harmful habits
- Diet counseling
- Splinting

4. Which surgical periodontal treatment do you carry out yourself in your clinic?

☐ Gingivectomy ☐ Flap Surgery ☐ Crown Lengthening

☐ Frenectomy/Vestibuloplasty ☐ Ridge Augmentation ☐ Free Gingival Autograft

☐ Others (Please Specify)

5. Do you refer patients to a periodontist for surgical procedures?

☐ YES     ☐ NO

A. If NO

   Please tick any of the following reasons:

- Carry out surgical procedures yourself
- Not satisfied with results of periodontal surgical treatment
- Have very few patients of periodontal disease who get motivated for periodontal surgery
- Others: (Please Specify)

   ^*^Phase I therapy includes scaling, root planing, dietary counseling and advising proper brushing technique and mouthwashes.

B. If YES

   a) How frequently do you call a periodontist for consultation/refer patients to a periodontist for surgical procedures

- Twice a week
- Once a week
- Once a month
- Others

   b) For which signs and symptoms of the patients do you consult a periodontist (Kindly rate according to frequency)

**Table:**

i.	Bleeding gums	[ ]
ii.	Presence of Periodontal pockets	[ ]
iii.	Mobile teeth	[ ]
iv.	Gingival enlargement	[ ]
v.	Periodontal Abscess	[ ]
vi.	Gingival Recession	[ ]
vii.	Other Mucogingival problems	[ ]

   c) What is the most common procedure performed by the Periodontist at your clinic (Kindly rate according to frequency)

**Table:**

i.	Flap Surgery	[ ]
ii.	Gingivectomy/Gingivoplasty	[ ]
iii.	Bone grafting/GTR	[ ]
iv.	Crown lengthening	[ ]
v.	Frenectomy/Vestibuloplasty	[ ]
vi.	Root coverage procedures	[ ]
vii.	Implants	[ ]

6. What is your opinion about the results of periodontal treatment

**Table:**

		Always	Occasionally	Never
a)	Stoppage of bleeding	[ ]	[ ]	[ ]
b)	Elimination of pocket	[ ]	[ ]	[ ]
c)	Reduction in mobility	[ ]	[ ]	[ ]
d)	Increased life span of teeth	[ ]	[ ]	[ ]
e)	Root coverage	[ ]	[ ]	[ ]
f)	Recurrence	[ ]	[ ]	[ ]

7. Do you recall your patients for maintenance therapy after the periodontal treatment

☐ YES     ☐ NO

A. If YES:

   Recall after:

- 1 Month
- 3 Months
- 6 Months
- In case of recurrence

8. What do you think are the factors responsible for the recurrence of the periodontal disease after the treatment

- Patient factors (Lack of maintenance and awareness)
- Periodontist factors (Lack of skill and time)
- General dentist factors (Lack of skill, chair-side time, motivation, awareness)

9. What is your opinion about the cost-effectiveness of the periodontal treatment

- Beneficial to all concerned
- Too costly for the patients

          Signature
